# Image-Guided Thermal Ablation as an Alternative to Surgery for Papillary Thyroid Microcarcinoma: Preliminary Results of an Italian Experience

**DOI:** 10.3389/fendo.2020.575152

**Published:** 2021-01-08

**Authors:** Giovanni Mauri, Franco Orsi, Serena Carriero, Paolo Della Vigna, Elvio De Fiori, Dario Monzani, Gabriella Pravettoni, Enrica Grosso, Marco F. Manzoni, Mohssen Ansarin, Gioacchino Giugliano

**Affiliations:** ^1^ Dipartimento di Oncologia ed Emato-Oncologia, Università degli Studi di Milano, Milan, Italy; ^2^ Divisione di Radiologia interventistica, Istituto Europeo di Oncologia, Istituto di Ricovero e Cura a Carattere Scientifico (IRCCS), Milan, Italy; ^3^ Scuola di Specializzazione in Radiodiagnostica, Università degli Studi di Milano, Milan, Italy; ^4^ Unità di Radiologia Clinico Diagnostica, Istituto Europeo di Oncologia, IRCCS, Milan, Italy; ^5^ Divisione di Psiconcologia, Istituto Europeo di Oncologia, IRCCS, Milan, Italy; ^6^ Divisione di Otorinolaringoiatria e Chirurgia Cervico Facciale, Istituto Europeo di Oncologia, IRCCS, Milan, Italy

**Keywords:** papillary thyroid microcarcinoma, radiofrequency ablation, laser ablation, thermal ablation, complications, recurrence, thyroidectomy

## Abstract

**Purpose:**

To report the results of our preliminary experience in treating patients with papillary thyroid microcarcinoma (PTMC) with image-guided thermal ablation, in particular estimating the feasibility, safety and short-term efficacy

**Materials and Methods:**

From 2018 patients with cytologically proven PTMC < 10 mm were discussed in a multidisciplinary team and evaluated for feasibility of image-guided thermal ablation. In case of technical feasibility, the three possible alternatives (i.e., image-guided thermal ablation, surgery, and active surveillance) were discussed with patients. Patients who agreed to be treated with image guided thermal ablation underwent radiofrequency (RFA) or laser ablation under local anesthesia and conscious sedation. Treatment feasibility, technical success, technique efficacy, change in thyroid function tests, side effects, minor and major complications, patients satisfaction and pain/discomfort perception during and after treatment, and disease recurrence during follow-up were recorded.

**Results:**

A total of 13 patients were evaluated, and 11/13 (84.6%) patients (9 female, 2 male, mean age 49.3 ± 8.7 years) resulted suitable for image-guided thermal ablation. All 11 patients agreed to be treated with image-guided thermal ablation. In addition, 3/11 (27.3%) were treated with laser ablation and 8/11 (72.7%) with RFA. All procedures were completed as preoperatively planned (technical success 100%). Technique efficacy was achieved in all 11/11 (100%) cases. Ablated volume significantly reduced from 0.87 ± 0.67 ml at first follow-up to 0.17 ± 0.36 at last follow-up (p = 0.003). No change in thyroid function tests occurred. No minor or major complications occurred. All patients graded 10 the satisfaction for the treatment, and mean pain after the procedure was reported as 1.4 ± 1.7, and mean pain after the procedure as 1.2 ± 1.1 At a median follow-up of 10.2 months (range 1.5–12 months), no local recurrence or distant metastases were found.

**Conclusions:**

Image guided thermal ablations appear to be feasible and safe in the treatment of PTMC. These techniques hold the potential to offer patients a minimally invasive curative alternative to surgical resection or active surveillance. These techniques appear to be largely preferred by patients.

## Introduction

Thyroid cancer is the most common malignant neoplasm of the endocrine system, representing the 3.1% of all cancers (1). In the last three decades, its incidence has increased worldwide due to the real increase onset and mostly to the increased detection (2). The most common malignancies arising in the thyroid gland are differentiated thyroid cancers, deriving from follicular cells. They include ﻿papillary thyroid carcinoma (PTC) and follicular thyroid carcinoma (FTC) (3). PTC is the most common subtype of thyroid malignancy accounting for 85% of thyroid cancer (4). The 50% of PTC is papillary thyroid microcarcinoma (PTMC), defined, according to the World Health Organization Classification of Thyroid Tumors, as a PTC less than 10 mm in maximum diameter (5). Most of these tumors remain clinically silent, have a bright prognosis, and a disease-specific mortality under 1% (2, 6).

Surgery is recommended as the first line-treatment for PTMC by current guidelines, the standard treatment modality being lobectomy (3, 7). However, surgery has some drawbacks, such as potential recurrent laryngeal nerve paralysis, hypothyroidism, hypoparathyroidism, need for lifelong medication, scarring, and risks connected with general anesthesia (6, 8). Moreover, it is not suitable for all patients because there are some ineligible patient due to systemic diseases, or others that refuse surgery (9, 10). Another therapeutic option, recommended by the American Thyroid Association, is active surveillance. Nevertheless, there is a low-risk of disease progression with this strategy (2%–6%) and this option is sometimes not well accepted by some patients (6, 9). Ito et al. analyzing data of 1,253 patients with PTMC under active surveillance, found that no one died or had distant metastasis during the observation period which ranged from 1.5 to 19 year (11). However, 58 (4.6%) had size enlargement, 19 (1.5%) had appearance of lymph-node metastasis, and 43 (3.5%) showed progression to clinical disease. Thus, patients with disease progression under active surveillance might require a much more aggressive surgical management than at the moment of the initial diagnosis.

Image-guided thermal ablations have been successfully applied in the treatment of several type of tumors, and have been recently proposed as a potential alternative to surgery also in patients with thyroid diseases (4, 12–14). These minimally invasive treatments, compared to surgical treatment have similar efficacy, fewer complications, better quality of life, and better cosmetic outcomes (15–17). These procedures allow precise delivery of the heat locally to the lesion, sparing the surrounding thyroid tissue, and thus minimizing the invasiveness of the treatment and the impact on thyroid function. However, even if first application of image-guided thermal ablations to treat a patient with PTMC has been reported in Italy in 2011 (18), experiences in literature are still limited, the majority of series having been reported by Chinese groups. At our center, we started to propose image-guided thermal ablation as a potential therapeutic option to patients with PTMC from 2018. Thus, this study aimed to report the results of our preliminary experience in treating patients with PTMC with image-guided thermal ablation, in particular estimating the feasibility, safety and short-term efficacy.

## Materials and Methods

Our Institutional Review Board approved this retrospective study. Patients included in this study provided written consent for anonymized data usage for research purpose. Institutional Review Board accepts this consent as informed consent for the present study.

Data of patients treated with image-guided thermal ablation for PTMC was retrieved from our prospectively collected database. All cases were discussed in our internal multidisciplinary tumor board. Image-guided thermal ablation was considered as a therapeutic option in patient with a single cytologically confirmed PTMC measuring <10 mm, with no contact with the thyroid capsule, and no ultrasound evidence of metastatic disease in the neck. All possible options, including image-guided thermal ablation, surgery or active surveillance were carefully discussed with the patient before treatment choice. Before treatment, a careful analysis by the interventional radiologist performing ablation was performed, evaluating the feasibility of the treatment, and the best path and technique for treating the patient. Exclusion criteria for image-guided thermal ablation were patients with clinically apparent multicentricity confirmed by ultrasound, with other type of thyroid malignancies, with cervical or distant metastasis revealed by ultrasound or other image techniques, with family history of thyroid cancer and/or history of radiation therapy, with contralateral vocal cord dysfunction, or with blood coagulation disorders.

### Ablation Technique

All procedures were performed with ultrasound (US) guidance under local anesthesia and conscious sedation. In all cases, a preoperative US evaluation was performed, to assess the nodule size and shape, proximity with critical structures, and to establish the best path to the target nodule. In case the nodule is located close to the periphery of the gland, or in a critical area, hydrodissection with the injection of sterile water was performed through a 21G needle. Contrast-enhanced US (CEUS) was always performed before ablation to assess nodule vascularization. At the end of the ablation CEUS was performed in order to immediately evaluate the result of the ablation. At our center, both radiofrequency ablation (RFA) and laser ablation are available for thyroid interventions. Decision of the best technique to be used is taken on a case by case basis by the interventional radiologist performing the procedure. For laser ablation one or two 21G introducer needles (depending from nodule size and shape) were inserted into the target nodule. Subsequently, a 300 micron quartz bare optic fiber was introduced into each introducer needle that was subsequently slightly withdrawn to expose the distal portion of the fiber. The optic fibers were connected to a multi-source laser system operating at 1.064 nm (EchoLaser X4, Elesta srl, Calenzano, Italy). A support planning tool device (ESI, Echolaser Smart Interface, Elesta srl, Calenzano, Italy), can be connected to a general US scanner and used for treatment planning. A fix power protocol (3 watts) was used, changing application time case by case, in order to maintain the power of each single application between 1,200 and 1,800 Joules.

For RFA an internally cooled, 18G electrode available with and 0.5 to 1.5-cm active tip (RFT-RFTS, RF Medical, Seoul, South Korea) was used. The free-hand technique was applied in all cases. Short ablations were performed with 30–50 watts power and eventually the electrode was repositioned in a different area in order to cover with subsequent ablations the entire volume of the target.

**Figure 1:**
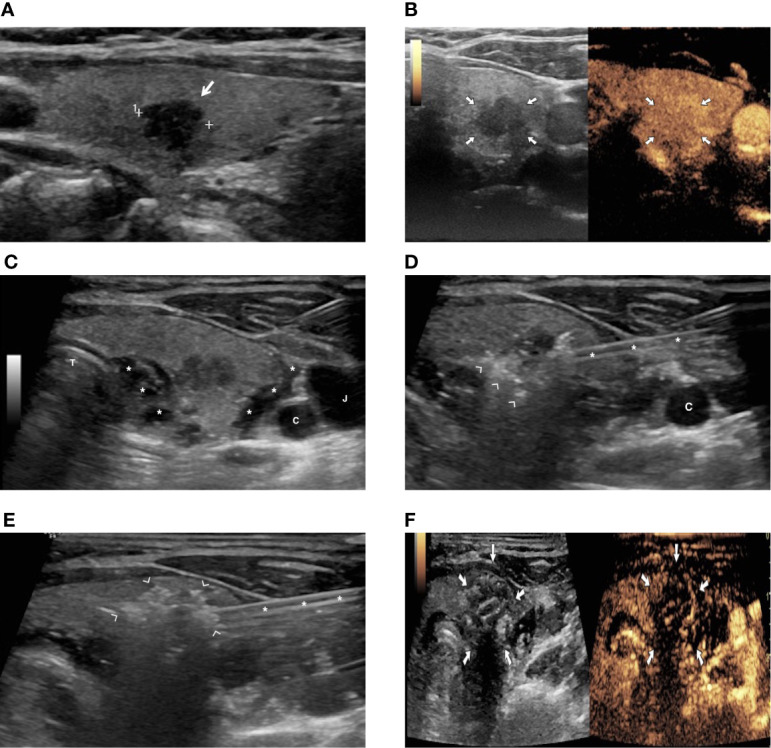
A patient with a PTMC treated with image-guided radiofrequency (RFA) ablation. **(A)** US image showing an 8-mm hypoechoic nodule in the right thyroid lobe (arrow). **(B)** Preoperative contrast-enhanced US demonstrating vascularization of thyroid lesion (arrows). **(C)** Hydrodissection with injection of 5% glucose solution (asterisks) to achieve separation of thyroid from vessels (C, carotid artery; J, jugular vein) and trachea (T). **(D)** Hyperechoic foci (arrowhead) due to gas formation during ablation around the thyroid nodule (asterisks = radiofrequency electrode). **(E)** Hyperechoic area (arrowhead) due to gas formation encompassing the whole thyroid nodule at the end of the ablation (asterisks = radiofrequency electrode). **(F)** Contrast-enhanced US image at the end of ablation, demonstrating lack of enhancement in the ablated area (arrows).

**Figure 2:**
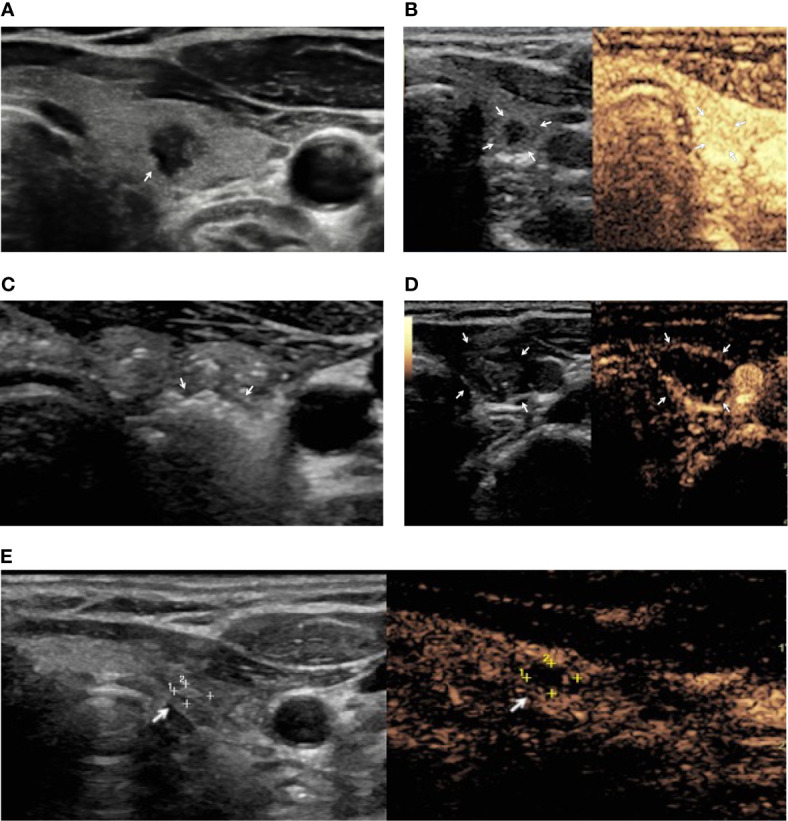
A female patient with PTMC treated with image-guided laser ablation. **(A)** US image demonstrating a 6 mm hypoechoic nodule with irregular margins (arrow). **(B)** Preoperative contrast US image demonstrating vascularization of thyroid lesion (arrows). **(C)** ﻿Hyperechoic areas (arrows) resulting from tissue heating and vaporization observed during laser ablation. **(D)** ﻿Contrast-enhanced US after ablation showing lack of enhancement in the treated area (arrows). **(E)** Nine-month follow-up: US image showing the residual ablated area decreased in size (arrow) and contrast-enhanced US demonstrating lack of enhancement in the ablation area (arrow).

Two cases of patients treated with RFA and laser ablation for a PTMC are shown in **Figures 1** and **2**.

### Study Endpoints

Treatment feasibility, technical success, technique efficacy, change in thyroid function tests, change in ablated area volume, side effects, minor and major complications, disease recurrence during follow-up and patients’ satisfaction and pain/discomfort during and after procedure were recorded. Standard definitions and reporting criteria were followed (19, 20).

Particularly, technical success was defined as the ability to complete the treatment that was preoperatively planned.

Technique efficacy was evaluated with US and CEUS at 6 weeks from the treatment.

Patients are followed with serum test evaluation of thyroid function, US and CEUS at 6 weeks, 6 months, and 12 months after treatment.

Patients were contacted by phone and were asked to grade on a scale from zero to ten the overall satisfaction regarding the procedure, the pain/discomfort perceived during the procedure and the pain/discomfort after the procedure.

## Results

A total of 13 patients were discussed for potential image-guided thermal ablation from 2018 and send to the interventional radiologist for evaluation of feasibility of the procedure. At the US examination, 2 cases demonstrated contact or invasion of the thyroid capsule, and so finally 11/13 (84.6%) patients (9 female, 2 male, mean age 49.3 ± 8.7 years) resulted suitable for image-guided thermal ablation. Patients’ characteristics are shown in **Table 1**. After discussion of all the three possible alternatives (i.e., image-guided thermal ablation, surgery, and active surveillance), all 11 patients agreed to be treated with image-guided thermal ablation. All patients had normal thyroid function before treatment.

**Table 1:** Characteristics of 11 patients with papillary thyroid microcarcinoma treated with image-guided thermal ablation.

Sex (Male/Female)	2/9
Age (years)	49.3 ± 8.7*
RCP cytology class (n)	
*Thy 4*	5
*Thy 5*	6
Nodule max diameter (mm)	7.9 ± 1.3*
Nodule volume (ml)	0.34 ± 0.51*
Power (Joules)	7,560 ± 3,786*
Ablation time (min)	5 ± 3.1*
Ablation volume at 1st FU	0.87 ± 0.67*
Ablation volume at last FU	0.17 ± 0.36*
Mean patient satisfaction (n)^#^	10 ± 0*
Mean pain during ablation (n)^#^	1.4 ± 1.7*
Mean pain after ablation (n)^#^	1.2 ± 1.1*

*mean ± standard deviation; ^#^on a scale from 0 to 10; RCP, Royal College of Patologists; FU, follow-up.

Of the 11 tumors treated, 3 (27.3%) were treated with laser ablation and 8 (72.7%) with RFA. All procedures were completed as preoperatively planned (technical success 100%). Technique efficacy was achieved in all 11/11 (100%) cases at 6 weeks control. 2/11 (18.1%) patients experienced a transient dysphonia beginning immediately after the local anesthetic injection around the thyroid capsule, before the ablation, that resolved few hours after treatment. 3/11 (27.3%) patients experienced mild discomfort and pain immediately after the procedure, which resolved in few days with only use of painkillers, and were regarded as side effects (19). No minor or major complications occurred. At a median follow-up of 10.2 months (range 1.5–12 months), no local recurrence or distant metastases were found. No change in thyroid function tests occurred. Ablated volume significantly reduced from 0.87 ± 0.67 ml at first follow-up to 0.17 ± 0.36 at last follow-up (p = 0.003). All patients graded 10 the satisfaction for the treatment, and mean pain after the procedure was reported as 1.4 ± 1.7, and mean pain after the procedure as 1.2 ± 1.1 (mean ± standard deviation).

## Discussion

The results of our preliminary experience show that image-guided thermal ablation can be safely applied in the treatment of PTMC, offering a potentially curative, minimally invasive treatment to patients in alternative to surgical resection or active surveillance.

Image-guided thermal ablations have been introduced in the treatment of cancer as an alternative to surgery in patients not suitable for surgical treatment several years ago (21, 22). Nowadays, indications have expanded, and image guided thermal ablations are applied in the treatment of a large variety of cancers (23–28), and in some cases represent the first suggested therapeutic option instead of surgery, as for the treatment of small hepatocellular carcinoma (29). Deriving from experience in other organs, and thanks to technological advancements with the creation of small dedicated ablative devices, image-guided thermal ablations have been applied also to the treatment of thyroid disease (30–35). Initially, image-guided thermal ablations have been used in the treatment of benign thyroid nodules, and then their use have been expanded to thyroid cancers not suitable for surgery and even metastatic disease (34, 36–40). Thus, in the large debate regarding the best management of indolent PTMC, were imaging derived overdiagnosis can drive a not negligible overtreatment, image-guided thermal ablations have been introduced with the rationale of providing a curative treatment to patients minimizing the invasiveness of treatment itself (41–44). Some series have been reported, mainly from Chinese and Korean authors, on the application of image guided thermal ablations in the treatment of PTMC with favorable results (45–47).

In our center, we included image-guided thermal ablation among the possible treatment option for PTMC starting from 2018. In our experience, 84.6% of patients sent for feasibility evaluation resulted finally suitable for treatment. This highlight the critical relevance of an accurate US evaluation before treatment, as selection of proper patient is of paramount importance for a successful procedure. Furthermore, of the suitable 11 patients, all agreed to be treated with image-guided thermal ablation over surgery or active surveillance. This highlights the potential impact of this technique for patients, which seems to prefer a treatment over active surveillance and is favorable to a minimally invasive approach. By taking into account results of a previous study assessing patient satisfaction for RFA or surgery for benign thyroid nodules (17), we might speculate that image-guided thermal would be preferred over surgery because of its lower invasiveness and higher cosmetic results. Simultaneously, ablation could be preferred over active surveillance because of anxiety and negative emotion likely related with receiving a cancer diagnosis without receiving active treatment and thus living with untreated cancer (48, 49). However, future research is essential to further assess patient preferences for image-guided thermal ablation, surgery, and active surveillance and identify main treatment attributes (e.g., clinical outcomes, aesthetic aspects, expected QoL, invasiveness, etc.) that might explain patient preferences for ablation over other therapeutic options for PTMC. Assessing patient’s perceptions and preferences for available treatments is becoming every day more important in decision making in oncology. Specifically, in recent years, there has been a shift toward a more patient-centered care and a growing emphasis on the relevance of involving patients in the clinical decision-making (50–53), as well in the evaluation of competing treatment options or health interventions (54, 55). For example, as highlighted by the P5 medicine approach (56, 57), each patient has a peculiar set of psychological and cognitive factors, such as preferences and needs and, as well as hopes, fear, beliefs and cognitive dispositions. The effective consideration of this psychological uniqueness and its integration with biological and clinical information is crucial to empower cancer patients and support their involvement in the clinical decision-making process as active decision-makers instead of merely passive recipients. Finally, the P5 medicine approach underlines the relevance of informing patients about all the available treatments in order to foster their participation in the treatment decision-making process. Thus, future efforts might be required to develop supportive and reliable tools that, such as patient decision aids (58, 59), provide patients with evidence-based health information about main therapies for PTMC.

From a clinical perspective, image-guided thermal ablation resulted feasible in all the selected cases, with a technical success of 100%. Also, no major or minor complications occurred in our experiences, while only a small percentage of patients referred side effects, which were mainly mild discomfort or pain after the procedure. This could be explained also with the large application of adjunctive procedures such as hydrodissection in our experience, which are crucial to preserve the surrounding structures, minimize the potential complications, and allow for an adequate safety margin for ablation (60–62). Immediate dysphonia after injection of local anesthesia around the thyroid capsule, can be due to the transient anesthesia of the recurrent laryngeal nerve, and is not regarded as a complication of the thermal ablation. Notably, no patients had change in their thyroid function after image-guided thermal ablation. Finally, during follow-up, no evidence of recurrence or disease progression was found in our patients. In a recent meta-analysis, thermal ablation techniques have shown efficacy in the treatment of PTMC, as noted by the pooled proportion of disappearance, the recurrence, and the volume reduction rate of PTMC which have been respectively of 57.6%, 0.4%, and 98.1% (9). Moreover, the pooled proportion of major complications was extremely low (0.7%), being represented by non-life-threatening voice change (9). In a recent series of 74 patients treated with RFA for 84 PTMC and followed for at least 5 years Cho et al., showed a disappearance rate of 100% at 60 months, no local tumor progression, no lymph node or distant metastasis, and no need for delayed surgery (47). Additional ablations were performed in 13 of 84 tumors. The major complication rate was 1.4% (1/74), and no procedure related death occurred.

Some limitations of our paper should be taken into account. First, this is a single center retrospective study, thus the number of patients is limited. However, this is the first experience in Europe on clinical application of image-guided thermal ablation in the multidisciplinary management of PTMC and highlights the potential of a larger application of these techniques in our countries. Second, the follow-up of patients is limited, in a disease with known slow progression. Thus, results on long-term clinical effectiveness cannot be derived from our results, which should be regarded as preliminary. Multicentric studies, evaluating larger samples and with longer follow-up are needed to better evaluate the potential role of image-guided thermal ablations in the treatment of patients with PTMC. Finally, we did not make comparisons with patients treated with surgery or who underwent active surveillance. As with thermal ablation, as with active surveillance, small foci of microcarcinoma or small central lymph node can be missed, it is of paramount importance to evaluate in the future long term follow-up in comparison with surgery.

In conclusion, image guided thermal ablations appear to be feasible and safe in the treatment of PTMC. These techniques hold the potential to offer patients a minimally-invasive curative alternative to surgical resection or active surveillance. Also, image-guided thermal ablations appear to be largely preferred by patients. Further studies on larger patient’s cohort are necessary to further address this issue.

## Data Availability Statement

The raw data supporting the conclusions of this article will be made available by the authors, without undue reservation.

## Ethics Statement

The studies involving human participants were reviewed and approved by European Institute of Oncology review board. The patients included in this study provided their written informed consent to participate in this study and for anonymized data usage for research purpose.

## Author Contributions

GM, FO, SC, PD, EF, DM, GP, EG, MM, MA, and GG contributed to the design and implementation of the research, to the analysis of the results, and to the writing of the manuscript. All authors contributed to the article and approved the submitted version.

## Funding

This work was partially supported by the Italian Ministry of Health with Ricerca Corrente and 5x1000 funds for IEO European Institute of Oncology IRCCS.

## Conflict of Interest

GM was consultant for Elesta SrL.

The remaining authors declare that the research was conducted in the absence of any commercial or financial relationships that could be construed as a potential conflict of interest.
